# Determinants of fractal motor activity regulation: insights from a large community-dwelling multi-ethnic population

**DOI:** 10.21203/rs.3.rs-10237495/v1

**Published:** 2026-07-06

**Authors:** Grégory Hammad, Emilie Seret, Christina Schmidt, Gilles Vandewalle

**Affiliations:** 1 GIGA-CRC Human Imaging, University of Liège, Liège, 4000, Belgium.; 2 Psychology and Neuroscience of Cognition Research Unit (PsyNCog), Faculty of Psychology and Educational Sciences, University of Liège, Liège, 4000, Belgium.

**Keywords:** Human, motor activity, fractal, rhythm, circadian, ultradian, multi-ethnic, ageing, actigraphy

## Abstract

**Background:**

Quantification of alterations in fractal motor activity regulation (FMAR) has the potential to improve early detection of Alzheimer’s disease. To improve the clinical relevance of such marker, our analysis aims to investigate potential determinants such as age, sex, physical activity and race and ethnicity in a large community-dwelling multi-ethnic cohort of older Asian, Black, Hispanic and White individuals.

**Methods:**

We analysed week-long actigraphy recordings from 1508 older adults (55 to 90 years) of the MESA Sleep dataset. Long-range correlations in activity levels, a marker of fractal regulation, were assessed via a scaling exponent extracted with a Detrended Fluctuation Analysis over two different time ranges. Bayesian linear models were used to compare scaling exponent values between ethnic groups and test their associations with various determinants, such as age, sex and physical activity levels.

**Results:**

We found opposite effects of age on FMAR at both short and long time scales, respectively (***β***_**Age**_ = **0.014**/***σ***_**Age**_, **95%** C.I. **[0.009**, **0.019]** and ***β***_**Age**_ **=** −**0.015**/***σ***_**Age**_, **95%** C.I. **[**−**0.026**, −**0.005]**), as well as differences between women and men at long time scales ($\beta_{\text{Sex}}^{\text{Men}}=+0.028$, **95%** C.I. **[0.012**, **0.044]**). We also confirmed that higher activity levels were linked to less degraded FMAR (***β***_**MDA**_ = **0.007**/***σ***_**MDA**_, **95%** C.I. **[0.003**, **0.011]** at short time scales and ***β***_**MDA**_ = **0.032**/***σ***_**MDA**_, **95%** C.I. **[0.024**, **0.041]** at long time scales). Finally, we observed that Black and Hispanic participants, as well as Asian participants, exhibiting lower values in scaling exponents at short and long time scales, respectively (***β***_**Black**_ **=** −**0.026**, **95%** C.I. **[**−**0.035**, −**0.017]**, ***β***_**Hispanic**_ **=** −**0.018**, **95%** C.I. **[**−**0.027**, −**0.009]** and ***β***_**Asian**_ **=** −**0.065**, **95%** C.I. **[**−**0.091**, −**0.039]**), compared to White participants.

**Conclusion:**

Using a large multi-ethnic cross-sectional dataset, our analysis provided support and clarified the interaction effect of age and sex on FMAR. It also confirmed, in humans, the influence of activity levels on FMAR observed in rodents and demonstrated for the first time that FMAR alterations are modulated by race and ethnicity.

## 1 Background

In mammals, physiological and behavioural signals, such as heart rate [1], breath [2], gait [3] or motor activity [4–8], exhibit non-linear complex regulation, yielding fractal, or scale-free, patterns that reflect the system’s adaptability. These patterns are altered in ageing but also in a series of disease conditions [9–11]. Indeed, alterations of fractal motor activity regulation (FMAR) patterns have been shown to be a predictor for Alzheimer’s disease (AD) and their reduction follows the disease progression [12–17]. In humans and rodents, these alterations have been linked to neuronal loss in the suprachiasmatic nucleus (SCN), the main circadian pacemaker, and in the dorsomedial part (DMH) of the hypothalamus [18–21], suggesting a mechanistic link between FMAR alterations and brain integrity loss in subcortical regions prone to neurodegeneration such as AD. The recent and rapid adoption, in the general population, of wearables that continuously monitor motor activity offers an opportunity to use FMAR as an additional marker for early AD risk assessment on a large scale. Nevertheless, in order to improve the clinical relevance of such information, researchers need to fully understand the factors that affect FMAR in community-dwelling older individuals, including but also beyond age and sex [12, 22].

FMAR patterns are degraded in both typical and pathological ageing but findings on sex differences in age-related FMAR alterations are inconsistent; no sex difference was found in a cohort of elderly individuals (mean age: 81 years) [15, 16] while women at younger age (mean age: 66 years) in preclinical AD stage specifically showed FMAR alterations [22], compared to men. Given that women are at a higher lifetime risk to develop AD than men [23], it is of primary importance to clarify the link between sex and FMAR.

Physical activity (PA) is a protective factor for health outcomes, including the risk of developing neurodegenerative diseases [24]. Yet, although FMAR inherently depends on PA, whether PA levels are related to FMAR is not established in humans. In mice, decreased PA was reported to cause alterations in FMAR and, critically, these alterations could be reverted by increasing PA, at both young and older age. It is crucial to understand the link between PA and FMAR in the context of an ageing population as the World Health Organization already recommends older adults to engage in physical exercises. One could potentially use FMAR to determine whether a physical activity intervention is beneficial or not for a given individual.

In the US, race and ethnicity have been linked to differences in health outcomes, including the prevalence of AD. Compared to White individuals, Black and Hispanic individuals are more likely to have AD and other types of dementia [25, 26]. Despite this increased risk, Black and Hispanic individuals are less likely to have the disease diagnosed [27, 28], exposing these minority groups to a higher burden of the disease. Furthermore, Asian individuals are under-represented in AD research and their estimate of AD prevalence might be under-estimated [29]. Finally, recent evidence highlighted race and ethnicity as a relevant factor for cut-off levels of biomarkers for AD diagnosis, such as plasma biomarkers [23]. However, it remains unclear how race and ethnicity influence FMAR.

Here, we studied various potential determinants of FMAR in a large multi-ethnic population. We analysed more than 1500 actigraphy recordings, covering a total of > 9000 days, from the MESA Sleep dataset [30] to asses fractal motor activity regulation. Using statistical modelling, we searched for associations between FMAR alterations and demographic factors such as age, sex and body mass index, as well as physical activity levels and ethnic origins. We hypothesized that FMAR alterations would increase with increasing age, be larger in women than in men and would be higher in participants with lower physical activity levels. Finally, we anticipated that non-White participants would have more altered FMAR compared to White participants.

## 2 Methods

### 2.1 Dataset and participants

The Multi-Ethnic Study of Atherosclerosis (MESA) is a longitudinal study of risk factors for cardiovascular disease in adults, aged above 45 years, with various racial and ethnic origins [30] (Asian, Black, Hispanic/Latino, and White). The initial baseline assessment was conducted between 2000 and 2002 in six different study centres across the United States of America (n=6814 participants).

Our analysis used data from 2237 MESA participants who were enrolled in the follow-up MESA Sleep study [30], which took place between 2010 and 2012. Participants contributed a week-long actigraphy recording of motor activity, a sleep questionnaire and an overnight ambulatory polysomnography (PSG) recording to the data collection. Data were accessed through the National Sleep Research Resource (NSRR) portal [31].

We excluded participants whose data were collected during a daylight saving time transition as well as participants reporting a work schedule including afternoon, night, split or irregular shift (metadata field: ‘wrksched5‘). A total of 1508 participants were finally selected for analysis.

### 2.2 Motor activity recordings

Motor activity was assessed with wrist-worn actigraphy recordings acquired with Actiwatch Spectrum devices (Philips Respironics) for seven consecutive days (median: 6.6 days, interquartile range, IQR: [6.5,6.9]). Activity data aggregated into 30-second epochs were scored as wake or sleep following a manual procedure documented by the NSRR [31]. Finally, activity data during wakefulness were selected based on this manual score and aggregated into 60-second epochs via summation for analysis purposes.

### 2.3 Fractal motor activity regulation (FMAR)

The fractal nature of motor activity was assessed through its scale-invariance (or self-similarity) property, using the *Detrended Fluctuation Analysis* (DFA) technique [1, 32]. Briefly, this method evaluates the scaling exponent (*α*) of the activity fluctuations (*F*) over time windows of increasing sizes (*n*): *F*(*n*) ∝ *n*^*α*^. In the case of a monofractal process, this scaling factor can be linked to the Hurst exponent, *H* [33, 34]. For *α* = 0.5, the signal is considered ”memoryless”; activity levels are temporally uncorrelated. For α > 0.5, the signal exhibits persistent long-term positive memory; upward fluctuations are more likely to be followed by upward fluctuations (and vice versa).

In practice, the scaling exponent, *α*, is derived by linearly fitting the natural logarithm of the activity fluctuations as a function of the logarithm of the time scales. In ageing and disease [12], the fluctuations are usually reduced, leading to a reduction in *α*. Since these reductions are larger at larger time scales, two different scaling exponents are usually defined; *α*_1_ for time scales below ~ 2h and *α*_2_ for larger time scales, potentially excluding a range of time scales between the two exponents. This difference is often summarized with a single variable: Δ*α* = *α*_1_ − *α*_2_.

In our analysis, we used DFA with a secondorder polynomial to detrend the signal. Fluctuations were computed over 80 different time scales, ranging from 5 minutes up to 1440 minutes (24h) and evenly distributed on a log scale. Scaling exponents, $\alpha_{\left[n_{1},n2\right]}$, were estimated with a linear regression of log *F*(*n*) over log(*n*), for *n* comprised between *n*_1_ and *n*_2_ minutes. This methodology relies on the assumption that the scaling exponent is constant over the range of considered time scales, meaning that despite the reduction in fluctuations, the functional relationship *F*(*n*) ~ *n*^*α*^, with *α* being independent of *n*, is conserved. To test this assumption, we computed a *local* scaling exponent, *α*_*L*_, at each time scale by restricting the linear fit of the natural logarithm of the activity fluctuations over the logarithm of the 3 preceding and 3 following time scales. This allowed us to test if the local exponents remained constant over a certain range of time scales and therefore could be summarized by a single exponent over this range.

### 2.4 Physical activity, race and ethnicity and other covariates

For each participant, physical activity was quantified by computing the mean daily activity (MDA) levels from actigraphy recordings. Race and ethnicity (R&E), as well as age and sex, were self-reported, and body-mass index (BMI) measured, during the study visit. Apnea-hypopnea index (AHI), defined as the number of apneas or hypopneas with 3% oxygen desaturation [35], per hour of sleep, was calculated using the overnight ambulatory PSG monitoring and categorized as normal (< 5 events/h), mild (< 15 events/h), moderate (< 30 events/h) and severe (> 30 events/h) [36].

### 2.5 Statistical analysis

Bayesian linear mixed models were used to test for statistical relationships between scaling exponents and health determinant factors such as age, sex, race and ethnicity, BMI, AHI category and MDA levels. The variables associated with age, BMI, and MDA levels variables were standardized (z-score) prior to statistical analysis.

Our model included sex, AHI category and race and ethnicity as categorical predictor variables and age, BMI and MDA levels as continuous predictor variables (fixed-effects). The general model structure was:

(1) $$\alpha_{\left[t1,t2\right]}=\beta_{\text{Intercep}}\;+\beta_{\text{Age}}*\text{Age}\;+\beta_{\text{Sex}}*(\text{Sex}==\text{Male})\;+\beta_{\text{Age|Sex}}*(\text{Age}|\text{Sex}==\text{Male})\;+\beta_{\text{BMI}}*\text{BMI}\;+\beta_{\text{MDA}}*\text{Mean}\;\text{daily}\;\text{activity}\;+\beta_{\text{AHI}}^{\text{Mild}}*(\text{AHI}\;\text{cat.}==\text{Mild})\;+\beta_{\text{AHI}}^{\text{Mod.}}*(\text{AHI}\;\text{cat.}==\text{Moderate})\;+\beta_{\text{AHI}}^{\text{Sev.}}*(\text{AHI}\;\text{cat.}==\text{Severe})\;+\beta_{\text{Asian}}*(\text{R}\&\text{E}==\text{Asian})\;+\beta_{\text{Black}}*(\text{R}\&\text{E}==\text{Black})\;+\beta_{\text{Hispanic}}*(\text{R}\&\text{E}==\text{Hispanic})\;+\epsilon$$

Female and White were used as reference categories for sex and race/ethnicity variables. As age, BMI, AHI and MDA levels were standardized, *β*_Intercep_ represents the estimated average scaling exponent for a 69-year old White woman with a BMI of 27.9, a normal AHI category and a MDA levels of 69.8. The parameter *β*_Age_ is the standardized age-effect coefficient for women, *β*_Sex_ the difference in scaling exponent for men compared to women, *β*_Age|Sex_ the additional age-effect coefficient for men, *β*_BMI_, *β*_AHI_ and *β*_MDA_, the coefficients associated to BMI, AHI category and MDA levels, *β*_Asian_, *β*_Black_ and *β*_Hispanic_ the estimated differences in scaling exponent, compared to White participants, for Asian, Black and Hispanic participants respectively and *ϵ* the matrix of residuals.

All statistical analyses were performed in Python 3.8.10, using Pandas (1.5.3) and Numpy (1.22.4) packages for data manipulation. Statistical Bayesian analyses were conducted with Bambi (v0.13.0) software [37]. Default weakly informative Gaussian priors were used and posterior distributions were estimated with a Markov Chain Monte Carlo (MCMC) technique, sampling 4 independent chains in parallel, with 20000 draws for each chain. Convergence and overlap between chains were considered acceptable if the potential scale reduction factor ($\hat{R}$) was smaller than 1.01. For each model parameter (*β*), the estimated posterior distribution was considered stable if its effective sample size was higher than 90% of the total number of draws (4 × 20000). Model adequacy was visually checked by comparing the data with the mean posterior predictive distribution. Finally, for each posterior distribution, we report its mean, standard deviation, and 95% highest density interval (HDI) as the credible interval (C.I.). Model parameter estimates were considered statistically significant if their 95% C.I. did not overlap with zero.

## 3 Results

### 3.1 Study participants

After applying our exclusion criteria, our analysis sample consisted in 1508 individuals. Their demographics and characteristics are reported in Table 1. Among them, the largest group consisted in White individuals (36.6%). Black and Hispanic/Latino individuals were the second and third largest group (27.5% and 24.1% respectively), while Asian individuals represented the smallest group (11.8%). The median age (69 years, IQR: [62, 77]) was found to be fairly similar among these different groups. However, the percentage of women varied across groups, ranging from ~ 53% in Asian and White participants, to ~ 58% in Asian and Black participants. Similarly, BMI exhibited small differences between groups: Asian participants had the lowest BMI (median: 27.4, IQR: [24.8, 31.9]) while Black, Hispanic/Latino and White had similar BMI (median: 27.8, IQR: [24.4, 31.5], median: 27.9, IQR: [24.7, 31.9] and median: 28.1, IQR: [25.2, 32.1], respectively). MDA levels also slightly differed between racial and ethnic groups: Asian participants had the highest activity levels, in 10^3^ counts per day (median: 108.7, IQR: [77.6, 135.2]), followed by Hispanic/Latino (median: 105.4, IQR: [80.3, 135.6]), while Black and White participants had the lowest levels (median: 98.3, IQR: [74.1, 124.0] and median: 96.9, IQR: [75.9, 126.1], respectively). Finally, AHI also exhibited small differences between groups: Black and Hispanic had the lowest numbers of events per hour of sleep (median: 17.5, IQR: [8.7, 29.4], median: 17.8, IQR: [9.7, 32.8], respectively) while Asian participants had higher numbers (median: 19.5, IQR: [10.5, 34.9]). The highest numbers were found for White participants (median: 20.4, IQR: [10.4, 35.0]).

### 3.2 Defining ranges of stable scaling exponents

**Fig. 1:**
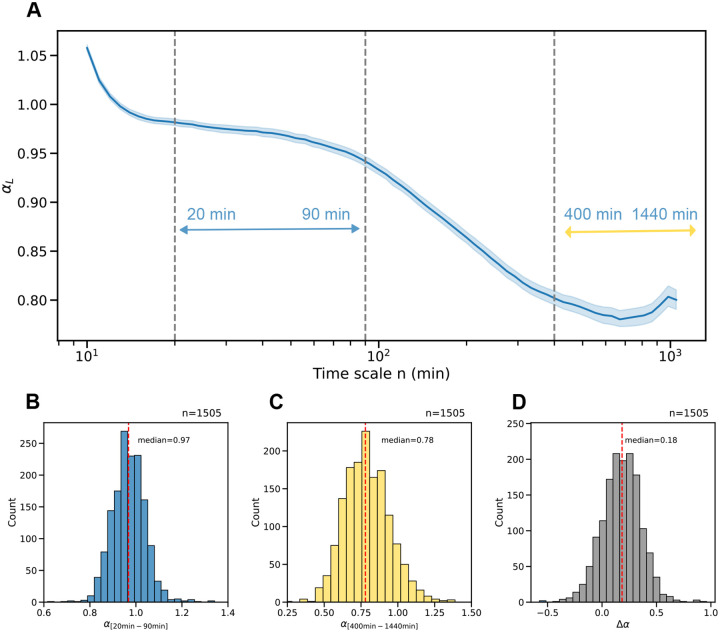
(A): Local scaling exponents (*αL*), averaged over the entire sample, as a function of time scales (n) in minutes. Confidence intervals at 95% on the average values are shown as shaded areas. Axes are displayed on a logarithmic scale and dashed lines are used to delineate the two time ranges ([20min-90min] and [400min-1440min]) where local scaling exponents are considered stable enough to allow a single scaling exponent to be computed. (B,C): Distribution of the scaling exponents at short and long time scales (*α*_[20−90]_ (B) and *α*_[400−1440]_ (C)). (D): Distribution of the difference between the two scaling exponents, Δ*α*. The median value of each distribution is depicted with a red dash line.

**Fig. A1:**
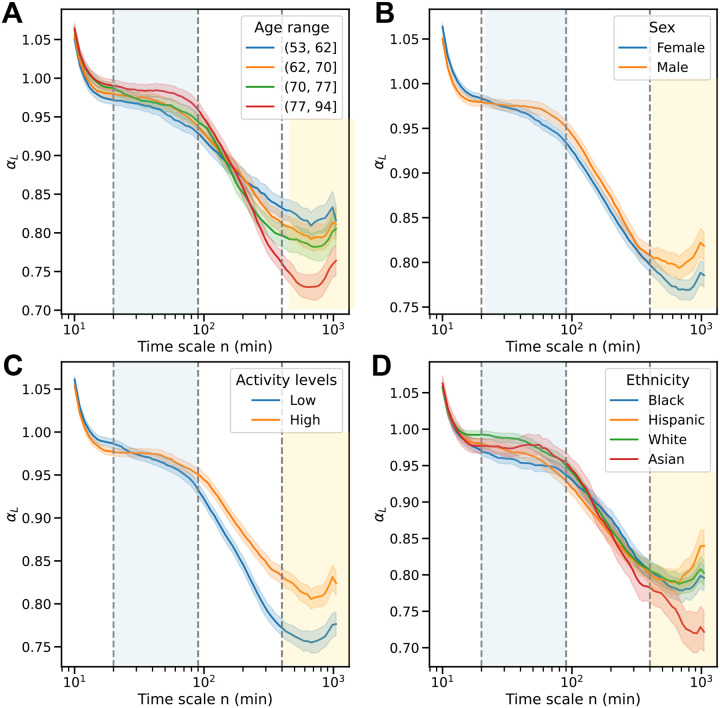
Local scaling exponents (*αL*), averaged over the entire sample, as a function of time scales (n) in minutes, stratified by age range (A), sex (B), mean daily activity levels (C) and race and ethnicity (D). Confidence intervals at 95% on the average values are shown as shaded areas. Axes are displayed on a logarithmic scale and dashed lines are used to delineate the two time ranges ([20min-90min] and [400min-1440min]) where local scaling exponents are considered stable enough to allow a single scaling exponent to be computed.

Local scaling exponents were first analyzed to determine if the time scale ranges commonly used in human studies of FMAR were appropriate for our dataset. For each participant, local scaling exponents were computed for each time scale (*n*) comprised between 10 min and 1024 min. The distribution of the mean of *α*_*L*_ over our sample, as a function of *n* are shown in Figure 1A. The scaling exponent does not decrease in a stepwise fashion but does decrease gradually with larger time scales. Nonetheless, similarly to previous FMAR analyses (e.g. [12, 20]), we identified two time scale ranges where the variations in scaling exponent are reduced, such that the scaling of the fluctuations can be modelled with a single exponent; the first range, from 20 to 90 minutes, is defined to strike a compromise between adequacy with our dataset and previous analyses. The second interval, however, ranges from 400 to 1440 minutes. This range starts at larger time scales than usually defined in other FMAR analyses [12, 15]. Finally, we cross-checked that these ranges were valid for sub-groups of our population stratified by the main factors of our analysis, namely age, sex, MDA levels and race and ethnicity. As shown in Figure A1, absolute *α*_[400−1440]_ values were influenced by these factors but remained stable over the two previously defined ranges.

In the following analysis, scaling exponents were computed over these two ranges, *α*_[20−90]_ and *α*[400−1440]. The difference, Δ*α* = *α*[20−90] − *α*_[400−1440]_, was also calculated to ease the comparison of our results with the existing literature on FMAR (e.g. [12, 16, 20]).

Scaling exponents, *α*_[20−90]_ and *α*_[400−1440]_, and their difference, Δ*α*, had unimodal distributions (Figures 1B–D) with a median value of 0.97 (IQR:[0.92, 1.02]), 0.78 (IQR:[0.68, 0.89]) and 0.18 (IQR:[0.07, 0.30]), respectively. Values of *α*_[20−90]_ and *α*_[400−1440]_ were in most cases greater than 0.5, confirming the presence of long-range positive temporal correlations in motor activity fluctuations, which is the distinctive feature of fractal regulation. Positive Δ*α* values also confirmed observations from previous studies; FMAR alterations (i.e. deviations of *α* from 1, the optimal value) are stronger at long time scales than at short time scales.

### 3.3 Influence of age, sex, physical activity and ethnicity on FMAR

**Fig. B2:**
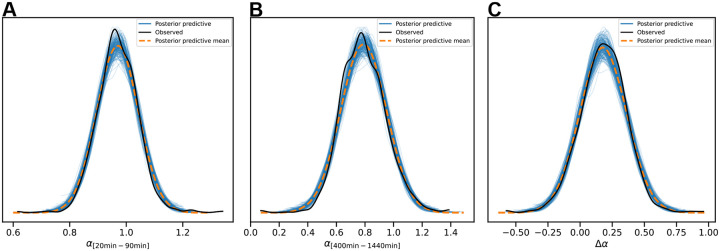
(A,B,C): Posterior predictive mean (dashed line) and data (solid line) distributions for *α*_[20−90]_, *α*_[400−1440]_ and Δ*α*.

Using our Bayesian statistical models, we analysed the effect of age, sex, MDA levels, AHI and ethnicity on daytime fractal regulation alteration at different time scales (Table 2). Models were validated by comparing posterior predictive distributions with data distributions (Fig. B2) for each outcome (*α*_[20−90]_, *α*_[400−1440]_ and Δ*α*).

#### 3.3.1 Opposite effects of age at short and long time scales on FMAR

**Fig. 2:**
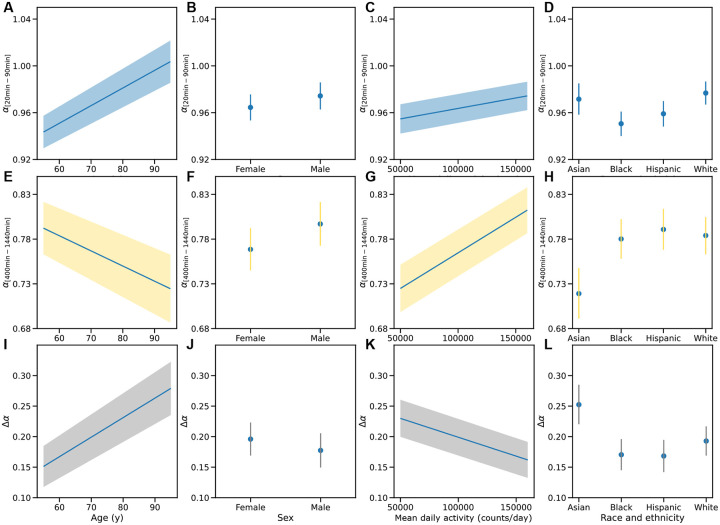
(A,B,C,D): Conditional adjusted prediction of the statistical model for *α*_[20–90]_ as a function of the participant’s age, sex, mean daily activity levels and race and ethnicity. (E,F,G,H): Conditional adjusted prediction of the statistical model for *α*_[400–1440]_ as a function of the participant’s age, sex, mean daily activity levels and race and ethnicity. (I,J,K,L): Conditional adjusted prediction of the statistical model for Δ*α* as a function of the participant’s age, sex, mean daily activity levels and race and ethnicity.

Age was differentially linked to FMAR as a function of the time scale range; testing for statistically significant relationship, we found that age was positively linked to *α*_[20−90]_ (*β*_Age_ = 0.014*/σ*_Age_, 95% C.I. [0.009, 0.019], Figure 2A), while negatively linked to *α*_[400−1440]_ (*β*_Age_ = −0.015*/σ*_Age_, 95% C.I. [−0.026, −0.005], Figure 2E). As expected, age was positively linked to Δ*α* (*β*_Age_ = 0.029*/σ*_Age_, 95% C.I. [0.017, 0.041], Figure 2I).

#### 3.3.2 Large differences of FMAR degradation between women and men at long time scales

As a main effect, sex had a statistically significant influence on FMAR, with men having a higher *α*_[20−90]_ (*β*_Sex_ = +0.009, 95% C.I. [0.002, 0.017], Figure 2B) and *α*_[400−1440]_ (*β*_Sex_ = +0.028, 95% C.I. [0.012, 0.044], Figure 2F), compared to women. This difference in magnitude of the effect of sex on FMAR between short and long time scales is reflected in an effect of sex on Δ*α*: *β*_Sex_ = −0.019, 95% C.I. [−0.037,−0.001], Figure 2J. In addition, we found that sex modulated the association between age and α_[20−90]_; indeed, the effect of age on *α*_[20−90]_, (*β*_Age_ = 0.014/*σ*_Age_, 95% C.I. [0.009, 0.019]) for women, was reduced by almost a half for men; (*β*_Age|Sex_ = −0.007/*σ*_Age_, 95% C.I. [−0.014, 0.000]).

#### 3.3.3 Higher activity levels are linked to less degraded FMAR

The effect on FMAR of the amount of physical activity, quantified by MDA levels, was investigated and found to have a statistically significant positive association with both *α*_[20−90]_ and *α*_[400−1440]_: *β*_MDA_ = 0.007*/σ*_MDA_, 95% C.I. [0.003, 0.011], Figure 2C and *β*_MDA_ = 0.032*/σ*_MDA_, 95% C.I. [0.024, 0.041], Figure 2G, respectively. The difference between *α*_[20−90]_ and *α*_[400−1440]_ in magnitude of these associations translated into a statistically significant negative association between MDA levels and Δ*α* (*β*_MDA_ = −0.025*/σ*_MDA_, 95% C.I. [−0.034, −0.016], Figure 2K).

#### 3.3.4 FMAR alterations are modulated by race and ethnic origins

Black and Hispanic participants had a statistically significant lower *α*_[20−90]_ than White participants (*β*_Black_ = −0.026, 95% C.I. [−0.035, −0.017] and *β*_Hispanic_ = −0.018, 95% C.I. [−0.027, −0.009], respectively, Figure 2D). For *α*_[400−1440]_, the value was statistically significantly lower for Asian participants (*β*_Asian_ = −0.065, 95% C.I. [−0.091, −0.039], Figure 2H), compared to White participants. Finally, these differences between ethnic origin of the participants, both in terms of magnitude and time scales, yielded opposite results for Δ*α*: compared to White participants (Figure 2L), statistically lower values of Δ*α* were observed for Black and Hispanic participants (*β*_Black_ = −0.022, 95% C.I. [−0.045, −0.000] and *β*_Hispanic_ = −0.025, 95% C.I. [−0.047, −0.001]), while higher values were measured for Asian participants (*β*_Asian_ = +0.058, 95% C.I. [0.028, 0.086]).

## 4 Discussion

The quantification of FMAR alterations with wearables opens the possibility to provide an additional tool for health status monitoring on a large scale. This requires, however, to systematically study the determinants of FMAR in the general population. Our study provides important insights to understand which factors influence FMAR in humans.

First, our analysis clarified a methodological aspect of the assessment of fractal patterns in motor activity with DFA. By computing a local scaling exponent, we demonstrate that the scaling behaviour of the fluctuations exhibits non-trivial patterns; while it seems possible to identify ranges of time scales with stable scaling exponents at both short and long time scales, where the scaling can be summarized with a single exponent, as previously observed [12], the dependence of *α*_*L*_ on the time scale is affected mainly by age and, to a lesser extent, by sex. This suggests that studies on FMAR might benefit from adapting the range of the time scales of interest to their target population demographics and might explain discrepancies observed in the determinants of FMAR between cohorts with different age ranges and sex compositions [15, 16, 22].

There are, at least, two underlying brain networks responsible for FMAR at different time scales ([18, 21]): the SCN and/or the DMH are key necessary nodes for fractal regulation of activity fluctuations at long time scales, while nodes for fractal regulation at short time scales remain elusive. As ageing goes along with a neuronal loss in subcortical regions including the SCN, it has been quite logically linked to reduced FMAR at large time scales [12]. Such reduction with age was confirmed in our sample. However, we observed a striking feature for FMAR at short time scales. The scaling exponent *α*_[20−90]_ was found to increase with older age. This increase was modulated by sex, with women exhibiting a steeper increase of *α*_[20−90]_ with age than men. While these results contrast with those reported by previous studies (e.g.[16]), this concomitant increase of *α*_[20−90]_ when *α*_[400−1440]_ is reduced has already been observed in rats after lesions of the SCN, during both light/dark and dark/dark cycles [18], demonstrating that this increase is not triggered by behavioural changes caused by light. As it seems unlikely that the neuronal integrity of the network responsible for FMAR at short time scale evolved positively with age in our sample, we hypothesize that the increase in *α*_[20−90]_ with age occur, at least partially, through the weakening of the interaction between the different networks responsible for FMAR. Similarly to the SCN, the central circadian pacemaker that does integrate peripheral feedbacks to fine-tune its circadian rhythm (e.g. [38]), the yet unknown network nodes regulating FMAR at short time scales could also integrate external cues, notably from the circadian system. In fact, a similar relationship between rhythms at long (circadian) and short (ultradian) time scales has already been reported in rodents: specific lesions to the subparaventricular zone (SPZ) of the hypothalamus, which receives projections from the SCN and in turn projects to the wake-promoting orexinergic lateral hypothalamus, lead to the concomitant loss of circadian rhythmicity and the emergence of ultradian oscillations, both in body temperature and motor activity, as if ultradian rhythms were unmasked by the weakening of the circadian signaling [39].

In our sample, we observed that FMAR was more altered in women than in men, as evidenced by lower values of both *α*_[20−90]_ and *α*_[400−1440]_ for women, especially at long time scale where the differences between women and men was ~ 2 times higher than the magnitude of the age effect, per decade of age. Our result confirmed previous reports of sex differences in FMAR, both for young and older participants [22, 40] but contrasted with results from [16]. However, differences in mean age and ratio of women (69.0 years and 55% in our case, compared to 80.9 years and 76% for [16]) might explain such differences. It is interesting to relate this result to the fact that the SCN is the central node of the network responsible for generating FMAR at long time scales. Indeed, compared to men, continuous loss of SCN volume with ageing has been reported to be more prominent in women [41]. In addition, the presence of amyloid plaque pathology during the preclinical phase of AD, as measured by amyloid positron emission tomography (PET) imaging, was associated with degraded FMAR but only for women [22]. Our results confirmed, in a large sample, previous observations about sex differences and therefore point towards the clinical relevance of FMAR, particularly in women which are at higher lifetime risk of AD than men.

In our data, activity levels were reduced with increasing age, as previously observed [42, 43] but we found that, in an older population but controlling for age effects, higher overall mean daily activity levels were associated with less altered FMAR. Similar results were observed in mice; the induction of low activity levels triggered FMAR alterations at long time scales that were reversible at all ages [44]. But, in contrast to mice, the association was seen in our sample both at short and long time scales. However, the magnitude of this association differed. Compared to the effect of age, the effect of the mean daily activity levels on *α*_[20−90]_ was about a half while this effect was at least twice larger for *α*_[400−1440]_ in our dataset. This result is interesting for two reasons; activity levels are known to modulate the circadian rhythm amplitude of the SCN outputs [45–47] but our results could also suggest that increasing activity levels is an effective way to restore the multiscale integrative function of the SCN. Obviously, we cannot rule out that a preserved multiscale temporal organisation of motor activity is necessary to efficiently maintain high activity levels. But, since low activity levels have been linked to cognitive decline and higher risks for developing Alzheimer’s diseases [24, 48], independently of age, this second hypothesis remains appealing from the interventional point of view and warrants further investigations. The second reason is the existence of an association between activity levels and FMAR at short time scales. The magnitude of this association is indeed reduced compared to the magnitude of the association at long time scales. However, notwithstanding a direct effect of activity levels on the system responsible for FMAR at short time scale, it can also be interpreted as an additional evidence for an interaction between networks operating at different time scales: activity levels influence *α*_[20−90]_ through the coupling between the SCN-driven network and the network responsible for generating FMAR at short time scales.

By demonstrating an association between FMAR alterations and risk of death, independent of age, sex, daily activity levels and chronic disease, Li and colleagues suggested that FMAR entails specific information about health status [16]. However, most samples used in FMAR studies so far overlooked a crucial health factor. In the U.S., the prevalence of AD is higher in racial and ethnic minorities [25, 26], with a lower probability of diagnosis and a diagnosis at a latter stage of the disease [27, 28], than in White population. The projected increase of the older population in the next decades, especially in racial and ethnic minorities [49], calls for a better understanding of these disparities and improved diagnosis tools. Our study reports disparities in FMAR between ethnic minorities. Overall, we found that FMAR was more altered for Asian, Black and Hispanic participants than for White participants, as assessed by a more reduced *α* value, at short or long time scales. However, ethnicity had a surprising influence on the time scales for which the FMAR degradation was more prominent, with Black and Hispanic participants being more affected at short time scales while the opposite was true for Asian participants. Another surprising aspect of race and ethnicity was the magnitude of the effect on FMAR: the difference in *α*_[400−1440]_ between Asian and White participants was more than twice the difference between women and men, while the difference in *α*_[20−90]_ between Black or Hispanic and White participants was two to three times larger than the effect of sex. This suggests that race and ethnicity is an important factor to consider for FMAR studies, although more studies are required to identify which risk factors, e.g. socio-demographic or genetic, contribute to our observed differences between racial and ethnic groups.

## 5 Limitations

The primary limitation of our analysis was the cross-sectional nature of the analysed dataset; we were not able to evaluate individual associations of change in FMAR with change in age or physical activity over time. Nonetheless, given the sample size of the MESA dataset, the large age range of the participants and the balanced woman/man ratio, compared to other datasets, we believe that our analysis provides valuable complementary results to existing FMAR analyses. Another limiting factor of our analysis arose from the absence of potential preclinical AD pathology assessment. It is impossible to exclude that the observed effects on FMAR were at least partially driven by individuals with preclinical AD neuropathology. Further studies are needed to evaluate the specificity of FMAR alterations in such individuals. Finally, our statistical analysis included several covariates related to individual’s health status such as activity levels, BMI and AHI. However, it lacked some socio-demographic factors that have been linked to health disparities between racial and ethnic groups in the US, such as education levels or house income. Future studies might include such factors to explain how they mediate the observed differences in FMAR.

## 6 Conclusion

To our knowledge, our study is the largest cross-sectional study on FMAR in a multi-ethnic population samples. Leveraging a sizeable age range (55 to 90 years old) and a balanced woman/man ratio, we provided robust evidence for effects of age and sex on FMAR, compatible with the expected age- and sex-dependent brain integrity loss in key regions of the hypothalamus. Besides, we reported evidence in humans for an effect of physical activity levels on FMAR, confirming results obtained in rodents, suggesting that the beneficial effect of increased physical activity on health status may be reflected in FMAR. Importantly, our results highlighted differences in FMAR alterations between ethnic minorities and white participants that are similar to known disparities in health status between racial and ethnic groups in the US, emphasizing the link between health status and FMAR. Overall, by clarifying the determinants of FMAR alterations, our study will help establishing fractal regulation as an additional marker for health monitoring that can easily be deployed on a large scale.

## Figures and Tables

**Table 1:** MESA Sleep participant demographics. Participant information are provided for the different ethnic groups for our study subsample. Numbers about age, BMI, MDA levels and AHI are provided as median and interquartile range.

	Race and ethnicity				
	All	Asian	Black	Hispanic/Latino	White
Participants (n, %)	1508	178 (11.68%)	414 (27.5%)	364 (24.1%)	552 (36.6%)
Age (year)	69.0 [62.0, 77.0]	68.5 [62.0, 76.0]	69.0 [63.0, 77.0]	70.0 [62.0, 77.0]	69.0 [63.0, 77.0]
Sex (women, %)	55.2	57.9	58.0	53.0	53.6
BMI (kg/m^2^)	27.9 [24.8, 31.9]	27.4 [24.8, 31.9]	27.8 [24.4, 31.5]	27.9 [24.7, 31.9]	28.1 [25.2, 32.1]
MDA (10^3^ counts/day)	100.5 [76.0, 129.0]	108.7 [77.6, 135.2]	98.3 [74.1, 124.0]	105.4 [80.3, 135.6]	96.9 [75.9, 126.1]
AHI (event/h)	18.8 [9.6, 33.2]	19.5 [10.5, 34.9]	17.5 [8.7, 29.4]	17.8 [9.7, 32.8]	20.4 [10.4, 35.0]

**Table 2:** Statistical analysis of FMAR scaling exponents. Results from a Bayesian linear model analysis: estimated mean, standard deviation and limits of the credible interval of the posterior distribution for fixed effects. Statistically significant results are reported in bold fonts. Age, BMI and mean daily activity variables have been standardized (*σ*_Age_ = 9.8 years, *σ*_BMI_ = 5.5 kg/m^2^ and *σ*_MDA_ = 40.8 · 10^3^ counts/day). Reference levels for categorical fixed effects: Female for Sex, White for Ethnicity and Normal for AHI category.

FMAR		Mean	Std.	95% HDI: lower limit	95% HDI: upper limit
α_[20min–90min]_	Intercept	**0.982**	**0.007**	**0.970**	**0.995**
	Age	**0.014**	**0.003**	**0.009**	**0.019**
	Sex[Male]	**0.009**	**0.004**	**0.002**	**0.017**
	Age—Sex [Male]	−**0.007**	**0.004**	−**0.014**	**0.000**
	BMI	0.001	0.002	−0.003	0.005
	Mean daily activity	**0.007**	**0.002**	**0.003**	**0.011**
	AHI (H3A) category[Mild]	−0.004	0.007	−0.017	0.009
	AHI (H3A) category[Moderate]	−0.005	0.007	−0.019	0.008
	AHI (H3A) category[Severe]	−0.007	0.007	−0.021	0.007
	Ethnicity[Asian]	−0.005	0.006	−0.018	0.007
	Ethnicity[Black]	−**0.026**	**0.005**	−**0.035**	−**0.017**
	Ethnicity [Hispanic]	−**0.018**	**0.005**	−**0.027**	−**0.009**
α_[400min–1440min]_	Intercept	**0.771**	**0.014**	**0.744**	**0.797**
	Age	−**0.015**	**0.005**	−**0.026**	−**0.005**
	Sex[Male]	**0.028**	**0.008**	**0.012**	**0.044**
	Age—Sex[Male]	−0.003	0.008	−0.018	0.012
	BMI	0.001	0.004	−0.008	0.010
	Mean daily activity	**0.032**	**0.004**	**0.024**	**0.041**
	AHI (H3A) category[Mild]	0.003	0.014	−0.024	0.031
	AHI (H3A) category[Moderate]	0.014	0.015	−0.015	0.042
	AHI (H3A) category[Severe]	0.028	0.015	−0.002	0.057
	Ethnicity[Asian]	−**0.065**	**0.013**	−**0.091**	−**0.039**
	Ethnicity[Black]	−0.004	0.010	−0.024	0.015
	Ethnicity[Hispanic]	0.007	0.010	−0.013	0.027
Δα	Intercept	**0.212**	**0.016**	**0.180**	**0.242**
	Age	**0.029**	**0.006**	**0.017**	**0.041**
	Sex[Male]	−**0.019**	**0.009**	−**0.037**	−**0.001**
	Age—Sex[Male]	−0.004	0.009	−0.022	0.013
	BMI	0.001	0.005	−0.010	0.011
	Mean daily activity	−**0.025**	**0.005**	−**0.034**	−**0.016**
	AHI (H3A) category[Mild]	−0.007	0.016	−0.038	0.026
	AHI (H3A) category[Moderate]	−0.020	0.017	−0.053	0.013
	AHI (H3A) category[Severe]	−**0.036**	**0.018**	−**0.070**	−**0.001**
	Ethnicity[Asian]	**0.060**	**0.015**	**0.030**	**0.089**
	Ethnicity[Black]	−**0.022**	**0.011**	−**0.045**	−**0.000**
	Ethnicity[Hispanic]	−**0.025**	**0.012**	−**0.047**	−**0.001**

## Data Availability

The data that support the findings of this study are available from the National Sleep Research Resource (NSRR) portal (https://sleepdata.org/datasets/mesa) but restrictions apply to the availability of these data, which were used under license for the current study, and so are not publicly available. Data are however available from the NSRR portal upon request.

## Appendix A Local scaling exponent distributions

The distribution of the mean of *α*_[400−1440]_ over our sample, as a function of n are shown in Figure A1, stratified by age, sex, mean daily activity levels and race and ethnicity. Mean daily activity levels above their median value (100, 6·10^3^ counts/day) were classified as “high” and “low” otherwise.

With increasing age, the local scaling exponent exhibited two different patterns: it increased at short time scales but decreased at long time scales, leading to a greater difference between local scaling exponents at short and long time scales. Sex differences were also observed: while the local scaling exponent for female participants was higher than for male participants at very short time scales (< 20 min), its decrease was steeper for females than for males up to a time scale of 50 − 60 min. Above that time scale, the decrease in local scaling exponents was identical for both sexes, although consistently lower for female participants than for male participants. Interestingly, these sex differences were quite similar to differences observed between participants with high and low mean daily activity levels, although the magnitude of the difference was stronger than the sex difference for time scales above 100 min and increased with increasing time scale values.

Finally, we observed differences between ethnic groups in the overall shape of the distribution of the local scaling exponent, with Black participants exhibiting lower exponents at short time scales (20 − 90 min) than White participants for example, while Asian participants exhibited the strongest decline at longer time scales. Nonetheless, all groups displayed fairly stable local exponents over the two ranges of time scales defined in this analysis, namely [20−90] min and [400−1440] min.

These observations highlighted both the difficulties and the importance to define appropriate time scale ranges for the assessment of stable scaling exponents.

## Appendix B Posterior predictive checks

Posterior predictive checks were performed to visually assess the ability of our statistical models to fit the data: expected outcome values obtained by randomly sampling posterior distributions were generated and their distribution compared to the original data distributions for *α*_[20−90]_, *α*_[400−1440]_ and Δ*α*. As show in Fig. B2, posterior predictive mean distributions were in agreement with the data for our three different outcomes.

## List of abbreviations

FMAR: Fractal motor activiy regulation
AD: Alzheimer’s disease
SCN: Suprachiasmatic nucleus
PA: Physical activity
MESA: Multi-Ethnic Study of Atherosclerosis
PSG: Polysomnography
NSRR: National Sleep Research Ressource
IQR: Interquartile range
DFA: Detrended fluctuation analysis
MDA: Mean daily activity
R&E: Race and ethnicity
BMI: Body mass index
AHI: Apnea-hypopnea index
MCMC: Markov Chain Monte Carlo
HDI: Highest density interval
CI: Credible interval
SPZ: Sub-paraventricular zone of the hypothalamus
PET: Positron emission tomography
